# A Polyhydroxyalkanoates-Based Carrier Platform of Bioactive Substances for Therapeutic Applications

**DOI:** 10.3389/fbioe.2021.798724

**Published:** 2022-01-05

**Authors:** Xu Zhang, Xin-Yi Liu, Hao Yang, Jiang-Nan Chen, Ying Lin, Shuang-Yan Han, Qian Cao, Han-Shi Zeng, Jian-Wen Ye

**Affiliations:** ^1^ Department of Chemical Engineering, Tsinghua University, Beijing, China; ^2^ Key Laboratory of Industrial Biocatalysis, Ministry of Education, Tsinghua University, Beijing, China; ^3^ Tsinghua-Peking Center of Life Sciences, Beijing, China; ^4^ School of Life Sciences, Tsinghua University, Beijing, China; ^5^ School of Biology and Biological Engineering, South China University of Technology, Guangzhou, China; ^6^ China Manned Space Agency, Beijing, China; ^7^ Guangdong Provincial People’s Hospital, Guangdong Academy of Medical Sciences, Guangzhou, China

**Keywords:** bioactive substances, therapeutic applications, polyhydroxyalkanoates, drug delivery, carrier platform, self-assembled PHA granules

## Abstract

Bioactive substances (BAS), such as small molecule drugs, proteins, RNA, cells, etc., play a vital role in many therapeutic applications, especially in tissue repair and regeneration. However, the therapeutic effect is still a challenge due to the uncontrollable release and instable physico-chemical properties of bioactive components. To address this, many biodegradable carrier systems of micro-nano structures have been rapidly developed based on different biocompatible polymers including polyhydroxyalkanoates (PHA), the microbial synthesized polyesters, to provide load protection and controlled-release of BAS. We herein highlight the developments of PHA-based carrier systems in recent therapeutic studies, and give an overview of its prospective applications in various disease treatments. Specifically, the biosynthesis and material properties of diverse PHA polymers, designs and fabrication of micro- and nano-structure PHA particles, as well as therapeutic studies based on PHA particles, are summarized to give a comprehensive landscape of PHA-based BAS carriers and applications thereof. Moreover, recent efforts focusing on novel-type BAS nano-carriers, the functionalized self-assembled PHA granules *in vivo*, was discussed in this review, proposing the underlying innovations of designs and fabrications of PHA-based BAS carriers powered by synthetic biology. This review outlines a promising and applicable BAS carrier platform of novelty based on PHA particles for different medical uses.

## Introduction

Thousands of patients lose their lives due to disease of tissue defects caused by accidents (Sadtler et al., 2016). Bioactive substances (BAS) treatment using small molecule drugs, proteins, RNA, cells, etc, was developed and proved to be an efficient theraputic solution for saving their lives (Farokhzad and Langer, 2009; Kamaly et al., 2016). Neverthless, the therapeutic effect is lmitted owning to the inherent characteristics of BAS, such as low bioavailability, cytotoxicity, poor physico-chemical stability and so on (Jain et al., 2013; Riley and Vermerris, 2017; Bizeau and Mertz, 2020; Nkanga et al., 2020). More importantly, the traditional BAS delivery systems also have some limitations such as over-dose and/or uncontrollable release of BAS, first-pass effect, etc. (Davoodi et al., 2018; Yu et al., 2020b; Laffleur and Keckeis, 2020; Nkanga et al., 2020), which would lead to untoward effect in human body. It is thus necessary to develop better BAS carrier systems to meet the requirements of such as sustainablly controlled release, biodegradable, biocompatible, etc. (Rambhia and Ma, 2015; Aguilar et al., 2019).

Recently, micro- and/or nano-particle carriers of BAS made from varied biomaterials are of growing interests for theraputic purposes because of their controllable biodegradation, weak immunogenicity, biocompatibility and accessibility (Pillai and Panchagnula, 2001; Kim et al., 2014; Ouazib et al., 2019). Currently, various biomaterials with micro- and nano-structures, for instance nanopaticles, microparticles, porous microspheres and nanofibers, have been designed for BSA encapsulation, allowing controllable release of BAS toward the targeted lesion locations (Liechty et al., 2010; Davoodi et al., 2018; Sung and Kim, 2020). Therefore, biomaterial-based carrier systems have been rapidly developed for diversified therapeutic applications, such as tissue engineering, oncotherapy, disease diagnosis, viccine protection and so on.

Polyhydroxyalkanoates (PHA) are a series of microbial synthesized biopolyesters accumulated as intracellular granules for carbon and energy storage in the presence of excessive carbon source and limited nitrogen and/or phosphorus sources (Choi et al., 2020a; Choi et al., 2020b; Zhang et al., 2020; Zheng et al., 2020). It is reported that many microorganisms including Gramnegative and -positive bacteria, such as *Ralstonia eutropha* and *Bacillus thermoamylovorans*, respectively, and even archaea like *Haloferax mediterranei*, are able to produce PHA (Chen and Jiang, 2017; Choi et al., 2020b; Moradali and Rehm, 2020). Notably, tailor-made PHA of diverse monomer structures, polymerization patterns and molecular weight can be obtained by genetically engineered microbes. Furthermore, physical and chemical modifications also offer alternative strategies to generate customized PHA with desired materials properties for high value-added uses (Kai and Loh, 2014; Li et al., 2016; Moradali and Rehm, 2020; Sharma et al., 2021). Thus, PHA was recently developed as novel-type BAS carriers for various theraputic applications due to the tunable mechanical properties, fully biodegradability and biocompatibility (Chen and Wu, 2005; Luo et al., 2019).

**FIGURE 1:**
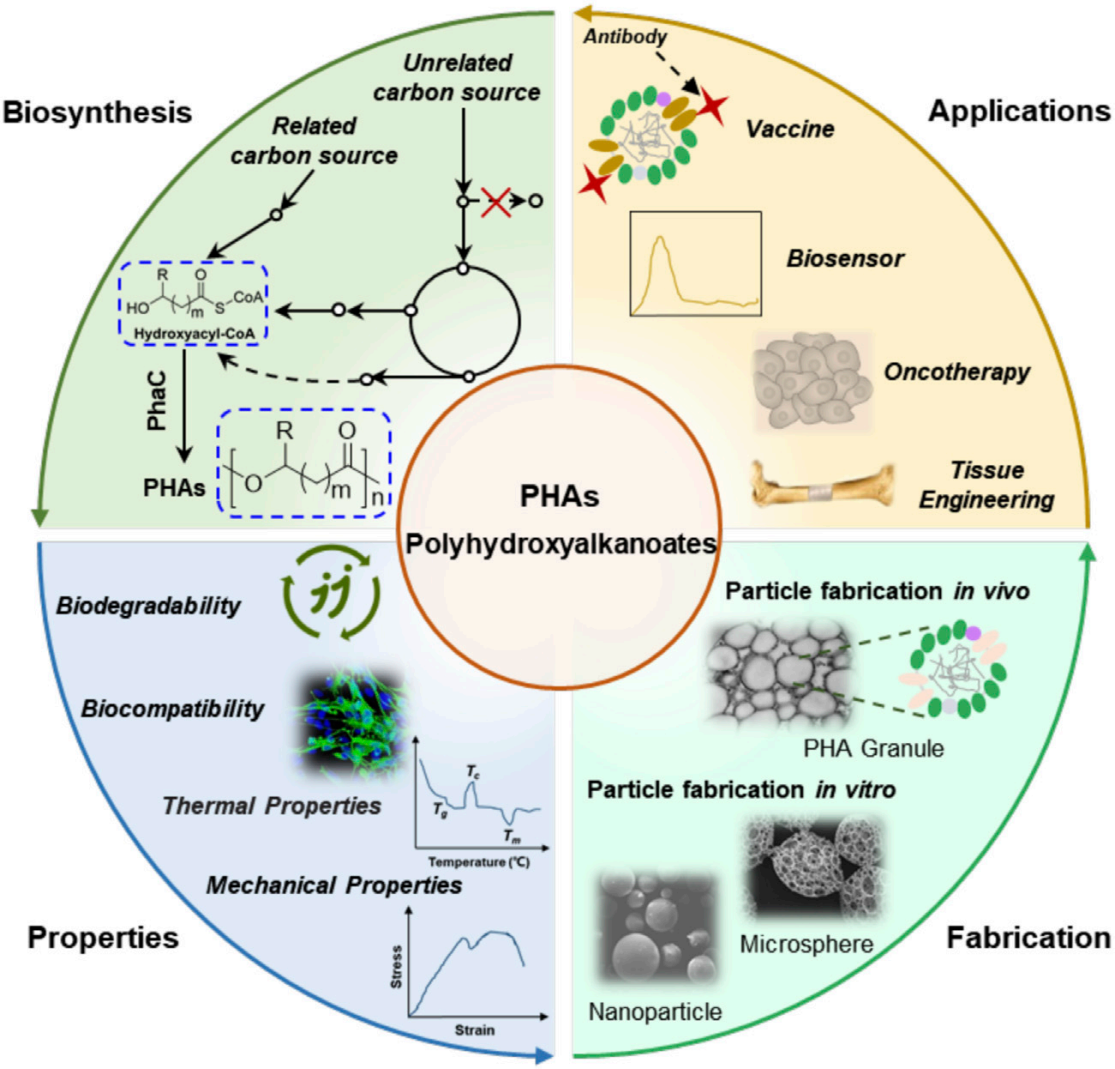
Overview of polyhydroxyalkanoates (PHA) from biosynthesis to therapeutic applications.

In this paper, we firstly summarized the typical synthesis pathways of PHA consisting of different monomers from related and unrelated carbon sources, resulting in different material properties, especially for thermal and mechanical properties. Then, we briefly introduce the fabrication strategies of micro- and nano-structure of PHA particles, including *in vitro* preparation of PHA particles and *in vivo* self-assembled PHA granules, which are widely used as BAS carriers. Finally, recent studies and advanced progresses of PHA-based BAS carrier systems are summareized and disscussed, giving an overview of perspectives and challenges of PHA-based carriers in pratical therapeutic applications (Figure 1).

## Biosynthesis of PHA

To date, although over 150 family members of PHAs have been reported, poly-3-hydroxybutyrate (PHB) is the first and most widely studied one discovered as inclusion bodies accumulated by a variety of microorganisms (Lee, 1996; Choi et al., 2020b). Three extensively studied enzymes, termed β-Ketothiolase (PhaA) encoded by *phaA*, NADPH/NADH dependent acetoacetyl-CoA reductase (PhaB) encoded by *phaB* and PHA synthase (PhaC) encoded by *phaC*, are involved in the last three biosynthesis steps of PHB by transforming acetyl-CoA into polymer. (Madison and Huisman, 1999; Steinbuchel and Lutke-Eversloh, 2003).

**FIGURE 2:**
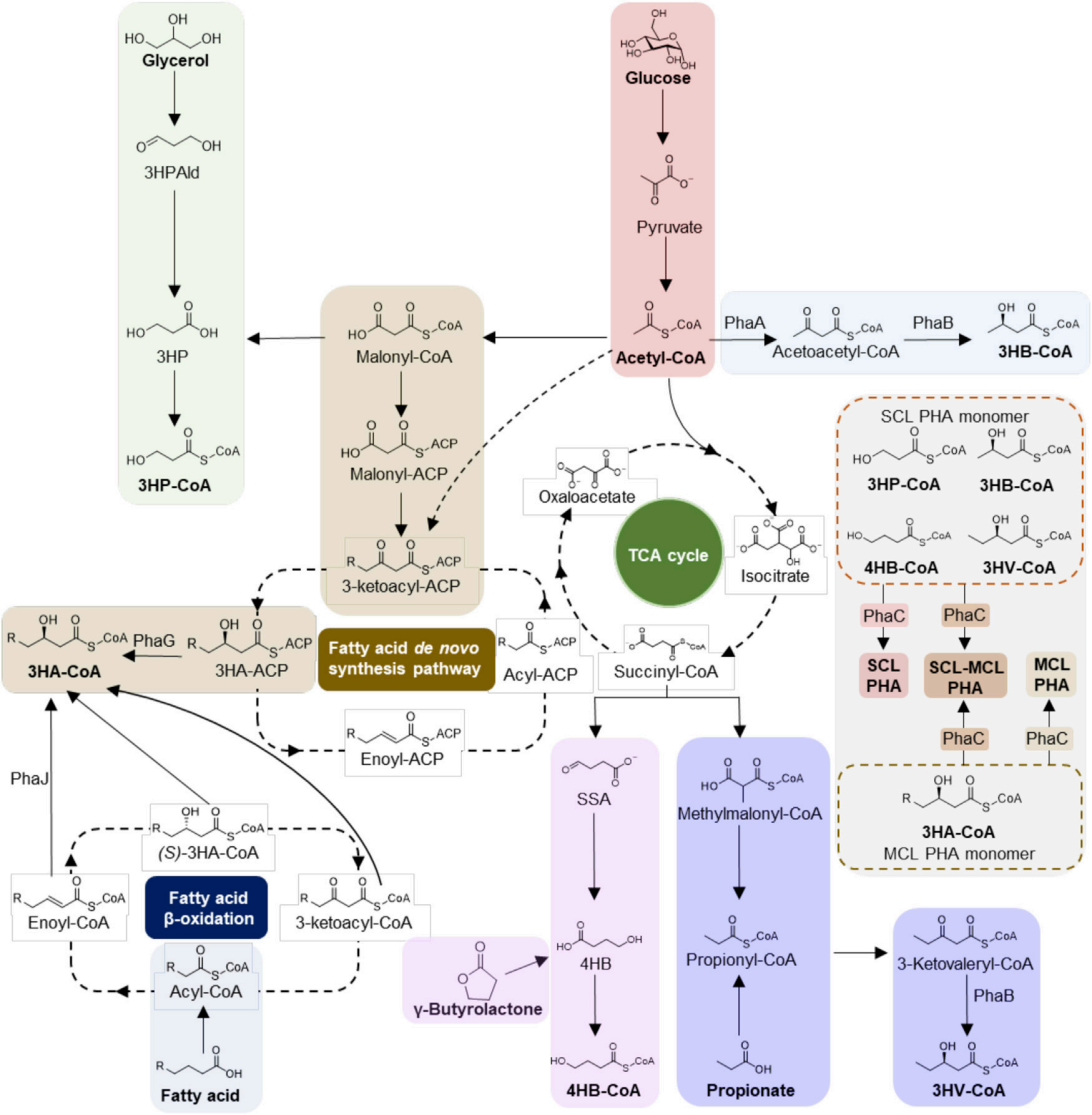
Biosynthesis pathways of SCL- and M/LCL-PHA. Metabolic pathways of short-chain length (SCL) PHA, medium/long-chain length (M/LCL) PHA and their copolymers (SCL-M/LCL) PHA are summarized. Specifically, SCL PHA indicates monomers containing only 3-5 carbon atoms (C3-C5), and monomers with over 6 carbon atoms are termed M/LCL PHA. The enzymes and metabolites shown are: PhaA, β-ketothiolase; PhaB, acetoacetyl-CoA reductase; PhaC, PHA synthase; PhaG, 3-hydroxyacyl-ACP-CoA transacylase; PhaJ, enoyl-CoA hydratase; 3HPAld, 3-hydroxypropionaldehyde; 3HP, 3-hydroxyproiponate; 3HA-ACP, 3-hydroxyacyl-ACP; SSA, succinate semialdehyde; 4HB, 4-hydroxybutyrate; 3HB-CoA, 3-hydroxybutyrate-CoA; 3HV-CoA, 3-hydroxyvaleryl-CoA.

However, the rapid development of synthetic biology enables efficient microbial cell factory engineering to create more pathways for non-PHB synthesis, resulting in various types of PHA production with tunable material properties, for instance homopolymers, random- and block-copolymers containing different monomer structures (Chen et al., 2016; Peregrina et al., 2021; Zhang et al., 2020), significantly expanding the diversity and availability of PHA. According to the carbon-atom number of monomers, PHA can be divided into two categories, namely short-chain length (SCL) PHA generally containing 3-5 carbon atoms (C3-C5), such as 3-hydroxybutyrate (3HB), 4-hydroxybutyrate (4HB), 3-hydroxyvalerate (3HV), 3-hydroxypropoinate (3HP), etc., and medium/long-chain length (M/LCL) PHA with over 5 carbon atoms (≥C6) (Choi et al., 2020b; Meng et al., 2014; Zhang et al., 2018b). Specifically, MCL PHA (C6-C14) and LCL PHA (≥C15) are generally accumulated by wild-type or recombinant *Pseudomonas* using fatty acids as carbon source (Meng et al., 2014; Nikel and de Lorenzo, 2018; Prieto et al., 2016); (Chen and Hajnal, 2015; Choi et al., 2020a; Li et al., 2021b; Singh and Mallick, 2009), while *Ralstonia eutropha, Halomonas bluephagenesis* TD01 and *Escherichia coli* have been developed to be promising workhorses for SCL PHA production with decades of research fundation and several industrial-scale successes. Herein, The major biosynthetic pathways of both SCL- and M/LCL-PHA by recombinant microbes from different structurally related and/or unrelated carbon sources (glucose, glycerol, fatty acids, etc.) are summarized in Figure 2.

In addition, enhanced PHA production can be achieved by knocking out bypasses and tuning the expression of target genes involved in different monomer synthesis pathways for sufficient supply of polymerization precursors. However, the PHA synthase with different specificity of substrates is the key enzyme to determine the diversity of monomer structure of PHA chains (Jung et al., 2010; Chen et al., 2016; Zhang et al., 2020). The molar ratio of each polymerization units can be easily moderated by controlling either the addition dosage of structure-related carbon sources or expression level of targeted synthesis pathways (Aldor and Keasling, 2003; Wang et al., 2019; Tan et al., 2020). A list of various PHA production by commonly used recombinant engineered chassis from different carbon sources was briefly summarized in Table 1.

**TABLE 1:** Representative production of PHA by different engineered chassis.

Engineered chassis	PHA products	Substrates	CDW (g/L)	PHA content (wt%)	Cultivation	Ref
*Escherichia coli*	PHB	Glycerol	12.23	2.7	Batch	de Almeida et al. (2011)
	P3HP	Glucose	5.35	18.41	Batch	Meng et al. (2015)
	PHBV	Glucose	1.6	46.1	Batch	Yee et al. (2012)
	MCL-PHAs	Molasses	4.05	75.5	Batch	Saranya and Shenbagarathai, (2011)
	PHBVDB	glycolate	0.61	12	Batch	Insomphun et al. (2016)
*Ralstonia eutropha*	PHBHHx	Poultry animal fats	3.3	82.8	Batch	Riedel et al. (2015)
	PHBHHx	Waste frying oil	2.4	86.0	Batch	Riedel et al. (2015)
	PHBHHx	Food waste	0.57	81.5	Batch	Bhatia et al. (2019b)
	PHBV	plant biomass hydrolysate	2.7–4.6	44–63	Batch	Bhatia et al. (2019a)
*Bacillus* spp.	PHBV	Sugarcane molasses	7.8	12.4	Batch	Das et al. (2019)
	PHBV	Waste cooking oil	3.5	85.0	Batch	Sangkharak et al. (2021)
	PHB	Whey	2.2	92.0	Batch	Israni et al. (2020)
	PHB	Starch	1.4	48	Batch	Singh et al. (2009)
*Halophiles*	PHBV	Glucose, gluconate	6.3	65	Batch	Chen et al. (2019b)
	P34HB	γ-butyrolactone, glucose	83	61	Fed-Batch	Chen et al. (2017)
	PHB, P34HB, PHBV	Starch	9.5	52	Batch	Lin et al. (2021)
	PHBHHx	hexanoic acid, glucose	2.63	29.31	Batch	Yu et al. (2020a)
	PHBHHxE	5-hexenoic acid, glucose	27.62	19.51	Fed-Batch	Yu et al. (2020a)
	PHB, PHBV	glucose, maltose, soluble starch, and propionate	8.82 (PHB), 33.45 (PHBV)	77.55 (PHB), 81.79 (PHBV)	Batch (PHB), Fed-Batch (PHBV)	Tao et al. (2021)
*Pseudomonas* spp.	MCL-PHAs	Cider by-products	3.7	14.5	Fed-Batch	Urbina et al. (2018)
	P (3HD)	Related carbon source (fatty acid)	2.06	19.42	Batch	Chung et al. (2013)
	P (3HDD)		2.8	85.13	Batch	
	P (3HTD)		1.99	37.69	Batch	
	P (3HB-*co*-3HN)		5.76	48.23	Batch	Li et al., 2021b
	P (3HB-*co*-3HUD)		8.91	65.96	Batch	

PHB, P3HP, PHBV, PHBVDB, PHBHHx, P34HB, PHBHHxE, P3HD, P3HDD, P3HTD, P (3HB-*co*-3HN), P (3HB-*co*-3HUD) and MCL-PHA are short for poly (3-hydroxybutyrate), poly (3-hydroxypropoinate), poly (3-hydroxybutyrate-*co*-3-hydroxyvalerate), poly (3-hydroxybutyrate-*co*-3-hydroxyvalerate-*co*-2,3-dihydroxybutyrate), poly (3-hydroxybutyrate-*co*-3-hydroxyhexanoate), poly (3-hydroxybutyrate-*co*-4-hydroxybutyrate), poly (3-hydroxybutyrate-*co*-3-hydroxy-5-hexenoate), poly (3-hydroxydecanoate), poly (3-hydroxydodecanoate), poly (3-hydroxytetradecanoate) poly (3-hydroxybutyrate-*co*-3-hydroxydecanoate), poly (3-hydroxybutyrate-*co*-3-hydroxyundecanoate) and medium-chain length polydroxyalkanoates, respectively; CDW, cell dry weight.

## Material Properties of PHA

### Biodegradability and Biocompatibility

PHA has been extensively studied in many medical uses due to its promising biocompatibility, (Chen, 2009; Zhang et al., 2018a; Luo et al., 2019). For *in vitro* tests, a variety of cell lines, including the ones, like stem cell, requiring strict cultural conditions, are able to grow on different PHA-based matrices of ignorable significance (Valappil et al., 2006). To be specific, human bone marrow mesenchymal stem cells (hBMSCs) could show better adhesion when grown on PHBVHHx film rather than TCPs and PLA films, which are FDA-approved implant materials (Hu et al., 2009). Besides, *in vitro* cultivation of mouse adipose stem cells (ADSCs) on plasma treated and untreated PHB and PHBV films displayed effect-free cell proliferation and excellent wound repairing capacity (Chang et al., 2018). More importantly, PHA-based biomaterials could help to determine the proliferation of stem cells, for instance, PHBVHHx can facilitate the osteogenic differentiation of MSCs (Novikova et al., 2008).

In addition, copolymer composed of 3-hydroxybutyrate and 4-hydrobutyrate (P34HB) with tunable elasticity and strength is a desirable biomaterial for the induced formation of elastin, which was developed as the substrate material of artificial blood vessels (Cheng et al., 2008). Several studies focusing on the fabrication and application of PHA-based artificial blood vessels have been reported to achieve required biocompatibility and mechanical properties, however, to date, no *in vivo* experimental verification has been conducted yet (Cheng et al., 2008). Furthermore, PHA have been demonstrated to be a desirable matrix for bone tissue engineering (Chen and Wu, 2005; Lim et al., 2017; Ray et al., 2019; Kalia et al., 2021). For example, PHBV scaffolds were used to repair spinal cord injury successfully (Ribeiro-Samy et al., 2013), PHBHHx exhibits outstanding performance in soft tissue fixation, such as tendon injuries and defects in a rat Achilles tendon repair model (Zheng et al., 2003), PHB scaffold displayed proven facilitation on the adhesion, proliferation and survival of adult Schwann cells while supporting axonal regeneration within the graft (Novikova et al., 2008). Moreover, it is reported that PHB conduits supported peripheral nerve regeneration (Young et al., 2002) in a rabbit common-peroneal-nerve injury model, and PHBHHx with better elasticity were successfully fabricated into porous nerve conduits for *in vivo* nerve repairing (Bian et al., 2009).

However, the unmodified PHA directly extracted from microbial cells with limited mechanical and thermodynamic performances generally can’t meet the demand of varied medical uses. Many approaches, such as chemical/physical modification, blending with other biomaterials, etc., have been developed to address these challenges (Raza et al., 2018; Sharma et al., 2021). For example, the chlorination of PHA could lead to significant changes of material property, from soft, sticky to crystalline, brittle and hard, which serves as a useful additive for polymer blending or further modifications (Arkin and Hazer, 2002). Besides, the chemical modified PHA provides desirable bioactivities for varied medical applications, such as antibacterial, bone tissue engineering, anticancer, etc. (Kai and Loh, 2014; Li and Loh, 2015; Raza et al., 2018). Notably, blending PHB with hydroxyapatite (HAP) and hydrogels is one of the widely used strategy to achieve significant improvements on compressive elastic modulus, maximum stress and osteoblast responses, including cell growth and alkaline phosphatase activity in both *in vitro* and *in vivo* culturing (Elmowafy et al., 2019).

Except the predominant biocompatibility, the fully biodegradable nature of PHA makes it become a popular player with better superiority of life cycle assessment (LCA) compared to the currently reported bio-based and/or degradable polymers. The degradation tests of PHA in laboratory-scale soil micro-ecosystem has demonstrated that the degradation rate is negatively correlated with the crystallinity of PHA (Volova et al., 2017). However, the PHA degradation rate could be accelerated when exposed to acidic or alkaline environments because of the constant formation of pore structures, which significantly increases the surface erosion area (Williams et al., 2013). Moreover, since the PHA polymers carrying carboxyl groups in the side chain showed higher degradation activity chemical modification of carboxyl group has been reported to be an efficient solution for elevating or preventing the PHA degradation process (Langlois et al., 2014).

Another prominent advantage of PHB and/or copolymers containing 3-hydroxybutyrate (3HB) unit is the beneficial degradation compound 3HB, which is demonstrated as an important component of mammalian ketone bodies able to provide energy sufficiently under extreme conditions, such as prolonged exercise, starvation, etc. (Newman and Verdin, 2017). More importantly, many studies have shown that 3HB has potential therapeutic effects on many diseases (Yao et al., 2021). 3HB and its derivatives could improve the learning and memory capabilities of mice, possibly through a signaling pathway requiring IFN-γ (PUMA-G) by increasing protein synthesis and gap junctional intercellular communication (Zou et al., 2009). Other neural benefits from 3HB in different diseases models, such as Alzheimer, Parkinson and other neurodegenerative diseases like epilepsy, are proved in many studies with effective therapy (Zhang et al., 2013; Paoli et al., 2014; Napolitano et al., 2020). Besides, the maintenance of 3HB level is also reported to resist inflammation and cardiovascular disease, for example, Zhang and her co-workers illustrated that daily nutritional supplementation of 3HB could attenuate atherosclerosis in mice (Zhang et al., 2021).

### Thermal Properties

Most PHA are partially crystalline polyesters. Generally, glass transition temperature (*T*_*g*_) for the amorphous phase, melting temperature (*T*_*m*_) for the crystalline phase and decomposition temperature (*T*_*d*_) are the three typical parameters used for thermal properties characterization (Laycock et al., 2013; Sudesh et al., 2000). As a result, thermal properties of different SCL PHA, MCL PHA and their copolymers are shown in Table 2 (Raza et al., 2018).

**TABLE 2:** Thermal and mechanical properties of PHA.

Types of PHA	Thermal properties		Mechanical properties			Ref
	*T*_*m* (°C)_	*T*_*g* (°C)_	Young’s modulus (MPa)	Tensile strength (MPa)	Elongation at break (%)	
Homopolymers of SCL						
PHB	178	4	3,500	43	5	Saito et al. (1996)
P4HB	61	−47	180.9	13.8	696.6	Meng et al. (2012)
P3HP	61.7	−24.4	2,889.3	33.8	497.6	Meng et al. (2012)
P3HV	104.2	−16.5	—	31.2	14	Yamane et al. (1996)
Copolymers of SCL						
P (3HB-*co*-36 mol% 4HB)	164	−11	—	4	400	Vigneswari et al. (2009)
P (3HB-*co*-75 mol% 4HB)	51	−45	—	16	526	Vigneswari et al. (2009)
P (3HB-*co*-20 mol% 3HV)	145	−1	—	20	50	Zhu et al. (2013)
P (3HP-*co*-25 mol% 4HB)	62.7	−31.3	14.5	1.7	962.9	Meng et al. (2012)
P (3HP-*co*-38 mol% 4HB)	63.5	−36.1	4.4	0.9	1,611.0	Meng et al. (2012)
Copolymers of MCL						
P (16 mol% 3HD-*co*-3HDD)	77.6	−32.5	103.1	5.2	88.3	Liu et al. (2011)
P (7 mol% 3HHx-*co*-3HO)	61.0	−37.8	1.2	7.4	346.3	Ma et al. (2009)
P (8 mol% 3HO-*co*-3HD)	69.4	−42.0	1.7	7.1	261.6	Ma et al. (2009)
P (44 mol% 3HD-*co*-3HDD)	74.9	−43.0	2.0	5.9	188.3	Ma et al. (2009)
Copolymers of SCL-co-MCL						
P (3HB-*co*-16.7 mol% 3HHx)	—	262.7	101.15	14.29	932.29	Yu et al. (2020a)
P (3HB-*co*-5.3 mol% 3HHxE)	149.34	269.4	294.66	21.04	22.86	Yu et al. (2020a)
P (3HB-*co*-22.80 mol% 3HN)	155.91	−1.34	59.64	4.09	89.32	Li et al. (2021b)
P (3HB-*co*-11.17 mol% 3HD)	154.71	−2.07	97.80	5.07	112.39	Li et al. (2021b)
P (3HB-*co*-15.67 mol% 3HUD)	156.38	−0.44	65.12	4.15	58.84	Li et al. (2021b)

*T*_*m*_, melting temperature; *T*_*g*_, glass transition temperature; 3HD, 3-hydroxydecanoate; 3HDD, 3-hydroxydodecanoate; 3HHx, 3-hydroxyhexanoate; 3HO, 3-hydroxyoctanoate; 3HHxE, 3-hydroxyhexenoate; 3HN, 3-hydroxynonanoate; 3HUD, 3-hydroxyundecanoate.

PHB, the most well studied PHA, has the highest *T*_*m*_ and *T*_*d*_ reaching up to 170°C and 200°C, respectively (Lee, 1996). Due to its non-sticky melting phase, PHB has been well studied and used for many industrial purposes, such as textiles (Jangra et al., 2018). However, the narrow range between *T*_*m*_ and *T*_*d*_ leading to instability during the thermal processing, which requires the copolymerization with other PHA monomers to improve the thermal stability when necessary (McAdam et al., 2020). The other two homopolymers, poly (3-hydroxypropionate) (P3HP) and poly (4-hydroxyalkanoate) (P4HB) having better thermal stability and higher crystallization rate, are of great competitiveness in many commercial utilizations (Meng et al., 2012; Williams et al., 2013; Andreessen et al., 2014).

Generally, the joint polymerization of 3HB and a second monomer including 3HV, 3HP and 4HB, is the most cost-effective strategy to obtain high yield production of PHA copolymers with enhanced thermal stability, representatives are shown in Table 2. Interestingly, compared to SCL-PHA homo- and/or copolymers, the MCL-PHA ones display lower crystallinity (Zinn and Hany, 2005). However, to some extent, low *T*_*m*_ value and crystallization rate of MCL PHA lead to limited uses. Therefore, several solutions, such as crosslinking, blending and grafting, have been developed for applicable uses, especially in therapeutic applications (Gopi et al., 2018; Choi et al., 2020a).

### Mechanical Properties

Owning to the high crystallinity of 60–80%, tensile strength of 43 MPa and Young’s modulus of 3.5 GPa, PHB shows similar unfavorable mechanical properties, such as brittleness and stiffness, in contrast to the conventional petrol-based polypropylene and polyethylene (Saito and Doi, 1994). Therefore, many efforts have been made to obtain flexible blending materials of PHB and other polymers such as P3HV, P3HP and P4HB (indicated by elongation at break), as well as elastic PHA copolymers composed of 3HB and other monomers with certain molar ratio, making PHA favorable in different added-value applications (Sudesh et al., 2000; Sharma et al., 2021).

Generally, MCL-PHA are crystalline elastomers exhibiting entirely different mechanical properties compared to SCL-PHA (Grigore et al., 2019). In previous studies, Li et al. has succeeded in producing tailor-made SCL-*co*-MCL PHA with complementary advantages of mechanics (Li et al., 2019; Li et al., 2021b). As shown in Table 2, the introduction of MCL-3HA units in PHB, forming P (3HB-*co*-MCL 3HA) copolymer, significantly increases the elongation at break of obtained copolymers, turning the brittle PHB into elastic PHA with ignorable variances in thermal properties (Li M. et al., 2021).

## Fabrication and Applications of PHA Particles

### Fabrication Methods

**FIGURE 3:**
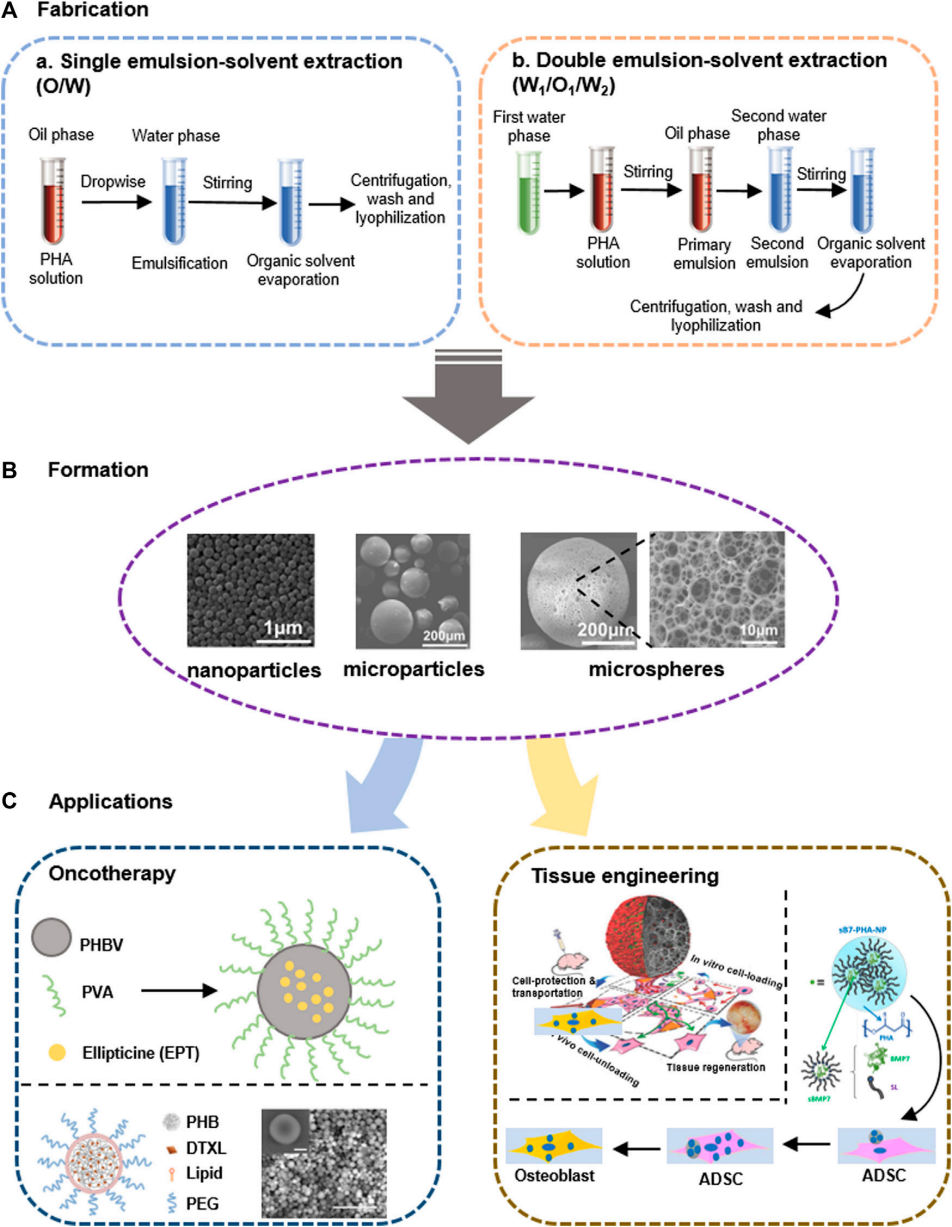
Fabrication and applications of PHA-based particles including micro-/nanoparticles and microspheres. **(A)** and **(B)** Fabrication methods of polymeric particles based on single and double emulsion-solvent extraction methods, respectively. **(C)** Therapeutic applications of PHA particles loaded with anti-cancer molecules ellipticine (EPT) and docetaxel (DTXL) for oncotherapy, as well as stem cells and growth factors like Bone Morphogenetic Protein 7 (BMP7) for tissue engineering. Abbreviations: PHBV, poly (3-hydroxybutyrate-*co*-3-hydroxyvalerate); PVA, polyvinyl alcohol; PHB, poly (3-hydroxyalkanoate); PEG, polyethylene glycol; ADSC, adipose-derived mesenchymal stem cell. Regenerated from (Di Mascolo et al., 2016), (Wei et al., 2018a) and (Chen et al., 2020) with permission.

Emulsification–solvent diffusion is one of the most widely used methods for the fabrication of PHA nanoparticles and microspheres (Figure 3A). Single emulsion-solvent extraction (O/W) is the firstly developed fabrication method. Firstly, polymer was dissolved in a water-immiscible organic solvent, such as dichloromethane (DCM), chloroform and ethyl acetate (Lu et al., 2011). Subsequently, BAS components like drugs were dissolved and dispersed into the pre-formed polymer solution to form oil phase (Li et al., 2021a). Followed by the dispersion of oil phase in an aqueous-phase solution, small oil droplets are obtained. With the removal of organic solvent under stirring condition, these oil droplets gradually solidify to form BAS-loaded PHA microspheres, which could be collected by ultracentrifugation and purified by distilled water washing to remove the impurities, such as surfactants, residual additives, etc. Finally, the lyophilized oil droplets are stored prior to use (Lu et al., 2011).

Furthermore, a double emulsion-solvent extraction (W_1_/O_1_/W_2_) method was developed for the encapsulation of water-soluble BAS (Figure 3A) (Gupta et al., 2017). Similarly, the first emulsion (W_1_/O_1_) was prepared by putting a BAS-dissolved aqueous solution into the pre-formed polymer (PHA) solution, then the second-round emulsification would be carried out in a new aqueous solution (W_2_) to generate W_1_/O_1_/W_2_ droplets. Following steps are the same with the preparation process of single emulsified ones (Vilos et al., 2013).

To increase the BAS loading capacity of PHA-based particles, pore-forming agents are employed to fabricate pore-enriched microspheres (Cai et al., 2013). Specifically, the pore-forming agents, such as ammonium bicarbonate allowing the gradual release of carbon dioxide and ammonia during its decomposition, would be firstly dissolved in the aqueous solution, and mixed with the pre-solved polymer solution to form emulsified PHA particles (Kim et al., 2006; Wei et al., 2018a; Wei et al., 2018b). Besides, sodium chloride (NaCl) was also used as pore-forming agent to obtain pore-structure microspheres after 2–3 times washing-out of NaCl. The porosity and pore size can be determined based on the supplemented concentration of NaCl. However, higher NaCl supplementation would reduce the mechanical properties of PHA-based microspheres (Chen et al., 2014a).

### Applications

#### PHA-Based Nanoparticles

PHA-based nanoparticles have been widely used for BAS delivery, especially therapeutic agents for oncotherapy purposes (Barouti et al., 2017; Li and Lim, 2018; Li and Loh, 2017), because PHA-based nanoparticles are desired carrier able to encapsulate highly toxic and water-insoluble chemotherapeutics for anti-tumor therapy (Figure 3C) (Michalak et al., 2017; Elmowafy et al., 2019). For example, ellipticine, a model anticancer drug of high toxic and poor solubility, was encapsulated in PHBV nanoparticles to increase the effectiveness of therapeutic treatment, showing improved inhibited activity of A549 cancer cell line (Masood et al., 2013). PHB-based nanocarrier was also used to deliver and control the release of docetaxel (DTXL), a potent anti-cancer drug, displaying higher drug loading efficiency and slower release rate in contrast to the commonly used PLGA (Di Mascolo et al., 2016). Taking this advantage, intracellular controlled-release of rhodamine B isothiocyanate (RBITC) using blend-PHA nanoparticles (PHB and PHBHHx) was also developed and achieved proven therapeutic effects by Xiong et al. (2010). More importantly, paclitaxel-loaded PHBV nanoparticles exhibits a 48-h window of toxicity protection allowing sufficient endocytosis of drug carriers by target cells, which can protect the anticancer agent against premature degradation with enhanced permeability and retention (EPR) effect (Vilos et al., 2013).

Similarly, PHBV nanoparticle encapsulated with photosensitizer (PS) compounds was developed by Pramual et al. to achieve enhanced photodynamic therapy (PDT) in cancer treatment (Pramual et al., 2016; Pramual et al., 2017). Moreover, PHA nanoparticles have been developed to function as targeting vectors and therapeutic agents carriers simultaneously for a wide range of local cells when necessary (e.g., simultaneous killing of tumor cells, tumor vasculature endothelial cells and suppressive T cells in a solid tumor compartment) (Li and Loh, 2017). Wu et al. developed a simple and safe nanoparticulate delivery system using PHBHHx copolymer for targeting different cell types mediated by the sub-cytotoxic level of polyethylenimine (Wu et al., 2014).

#### PHA-Based Microspheres

Due to the convenient local injection without adverse reactions, PHA microspheres have great potential in antitumor therapy and tissue regeneration (Figure 3C) (Papaneophytou et al., 2019; Yuan et al., 2021). It is reported that rubomycin deposited in PHA microspheres exhibited pronounced improvement of antitumor activity by inhibiting the proliferative activity of Ehrlich ascitic carcinoma, and finally improved the survival of mice with tumors. And local injection is a perfect option for this drug dosage control (Shishatskaya et al., 2008). High encapsulation efficiency and sustainable release of antibiotics, such as tetracycline and gentamicin, deposited in PHA microspheres could also be achieved with therapeutic effects in corresponded disease models (Sendil et al., 1999; Zhang et al., 2012). For example, Zhang et al. prepared PHBHHx-based microspheres with a capillary-size diameter of 5–10 μm, carrying adenoviruses fused with a green fluorescence protein for therapeutic study (Zhang et al., 2012). The PHA-protein complex was then injected into the pancreas of mice via the celiac trunk. Five days after the surgery, approximately 40% of endothelial cells in the pancreas were labeled, while the other cells in the islet were merely detectable. Therefore, this efficient pancreatic capillary targeting implant based on PHA microsphere-adenoviral complexes are of great potential and values in capillary-related diseases treatment in the coming future (Zhang et al., 2012).

To avoid large open surgery using scaffold transplants, small-sized cell carriers have been developed and employed to repair complicated tissue defects (Wei et al., 2018a; Yuan et al., 2021). Therefore, PHA microsphere is an excellent choice due to its tunable mechanical properties and fully biocompatible nature. Wei et al. has successfully manufactured highly open porous microspheres (OPMs) of 300–360 μm in diameter, serving as injectable carriers harboring proliferating stem cells (Figure 3C) (Wei et al., 2018a). In contrast to PLA microspheres, PHA OPMs show better biocompatibility of supporting the proliferation and differentiation of hMSCs during allogeneic bone formation in mice and *in vitro* test. Being like a micro-Noah’s Ark for cells, PHA microspheres help to transport the *in vitro* proliferated cells into the defect tissues for tissue regeneration (Wei et al., 2018a). Recent efforts focused on fabricating PHBV-based mesoporous bioactive glass nanoparticles (MBGN) loaded with different concentrations of cinnamaldehyde (CIN) shows effective antimicrobial activity and rapid hydroxyapatite formation in a simulated body fluid model, demonstrating the great potential of PHA particles used as antibiotic-free material in bone defects repair (Chotchindakun et al., 2021). Studies of PHA-based particles for medical uses are collected and shown in Table 3.

**TABLE 3:** Applications of PHA-based particles including micro-/nanoparticles and microspheres.

	PHA	Particle size	BAS	Applications	Ref
Nanoparticle	PHB	201.5 nm	docetaxel	Anti-cancer	Di Mascolo et al. (2016)
	PHB	160.6 nm	epirubicin	antibacterial	Perveen et al. (2020)
	PHB	199.3 nm	Sorafenib/doxorubicin	Anti-cancer	Babos et al. (2020)
	PHBV	169.0–211.2 nm	photosensitizer	photodynamic therapy (PDT) in cancer treatment	(Pramual et al., 2016; Pramual et al., 2017)
	PHBV	152.3 nm	epirubicin	antibacterial	Perveen et al. (2020)
	PHBHHx	100 nm	docetaxel	melanoma solid tumors	Luo et al. (2018)
	PHBHHx	180 nm	etoposide	Anti-cancer	Kılıçay et al. (2011)
	P34HB	150 nm	docetaxel	Anti-cancer	Faisalina et al. (2020)
	PHBVHHx	95.7 nm	azathioprine	systemic lupus erythematosus and reduced side effects	Hu et al. (2020)
Microsphere	PHB	4–92 μm	dipyridamole	antithrombotic	Bonartsev et al. (2007)
	PHB	6.98 μm	curcumin	antioxidant, anti-inflammatory, and antimicrobial	Aguilar-Rabiela et al. (2020)
	PHBV	322–530 μm	tetracycline	periodontal diseases	Sendil et al. (1999)
	PHBV	6.1–12.5 µm	cinnamaldehyde	antimicrobial	Chotchindakun et al. (2021)
	PHBHHx	40–200 μm	triamcinolone acetonide	acute posterior segment inflammation associated with uveitis	Bayram et al. (2008)
	PHBVHHx	300–360 μm	hMSCs	injectable carriers of stem cells	Wei et al. (2018a)

PHB, poly (3-hydroxybutyrate); PHBV, poly (3-hydroxybutyrate-*co*-3-hydroxyvalerate); PHBHHx, poly (3-hydroxybutyrate-*co*-3-hydroxyhexanoate); P34HB, poly (3-hydroxybutyrate-*co*-4-hydroxybutyrate); PHBVHHx poly (3-hydroxybutyrate-*co*-3-hydroxyvalerate-*co*-3-hydroxyhexanoate); hMSCs human bone marrow mesenchymal stem cells.

## Novel-Type Carriers Based on *in Vivo* Self-Assembled PHA Complex

**FIGURE 4:**
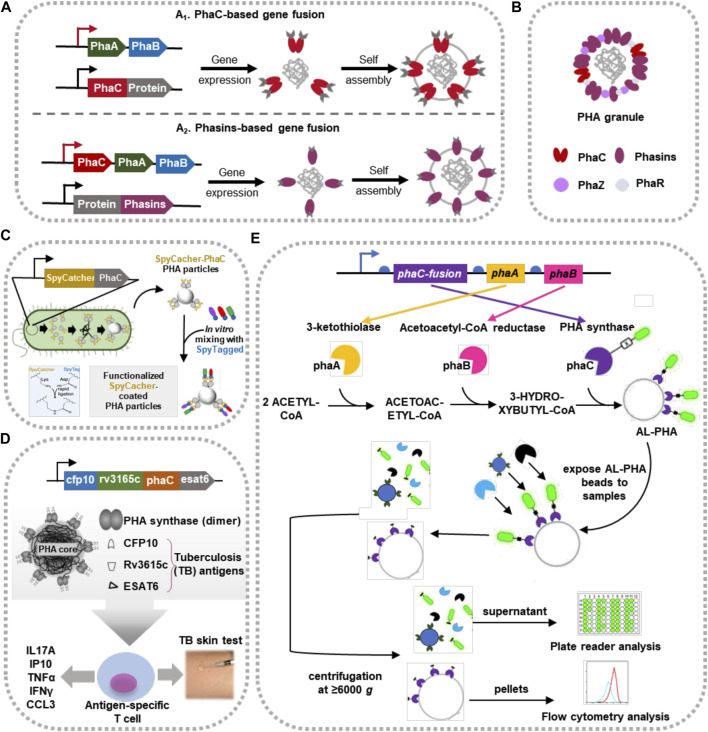
Fabrication and applications of PHA-based BAS carriers. **(A)** Functionalization of self-assembled PHA granules using PhaC- (A_1_) and phasins- (A_2_) based fusion approach. **(B)** Structural schema of a natural synthesized PHA-protein complex. **(C)** Functionalized PHA particles coated with Spy-catcher tag to form immobilized biocatalysts scaffold. Regenerated from (Wong and Rehm, 2018) with permission. **(D)** TB antigens modified PHA particles as vaccine for skin test. Regenerated from (Parlane et al., 2017) with permission. **(E)** Schematic diagram of AL-PHA system (Advanced proteoLytic detector PolyHydroxyAlkanoates). Regenerated from (Kelwick et al., 2021) with permission.

PHA-protein complex is generally comprised of an amorphous hydrophobic PHA granule coated with PHA-associated proteins, namely PAPs, including PHA synthase (PhaC), phasins (e.g., PhaP and PhaF), PHA depolymerase and other structural and/or regulatory proteins (Figures 4A,B) (Parlane et al., 2017). Specifically, PhaC and the noncatalytic protein phasins, which generally consist of a hydrophobic domain associated with the surface of PHA granules and a hydrophilic/amphiphilic domain exposed to the intracellular cytoplasm (Mezzina and Pettinari, 2016), are predominant candidates for BAS loading to fabricate novel-type nano-carriers (Figure 4A) (Wong et al., 2020). Recently, many efforts have been made to develop novel-type BAS-carriers based on self-assembled PHA complex loaded with different immobilized biocatalysts scaffold, vaccines and biosensors for different therapeutic purposes (Figure 4).

### Immobilized PHA Scaffold

Redesigning PAPs coated on intracellular PHA granules on a translational level enables the one-step immobilized display of target enzymes on PHA-based scaffold (Parlane et al., 2017). Specifically, different heterogenous proteins of interest could be fused to the N- or C-terminus, or both termini of PAPs (mainly PhaC via covalent interactions and phasins via physical adsorption) for fusion expression (Dinjaski and Prieto, 2015). Compared to the dissociated enzymes, the immobilized enzymes coated on PHA scaffold shows enhanced stability, reusability and catalytic performance (Wong et al., 2020). Therefore, a generic and modularized immobilization system was constructed by combining PAPs-based display system and SpyCatcher/SpyTag connector, providing a versatile plug-and-play mode of immobilized PHA scaffold (Figure 4C) (Wong and Rehm, 2018). The SpyCatcher-PHA immobilization platform displays outstanding thermal and pH stability in many application assessments when using eukaryotic therapeutic enzymes, industrial added-value proteins, etc., as target objects (Wong and Rehm, 2018).

### Vaccines and Biosensors

PAPs coated PHA inclusions are naturally self-assembled complex stored inside the bacterial cells, which has developed to be a versatile vaccine platform for producing synthetic multivalent vaccines of low cost (Gonzalez-Miro et al., 2019; Wibowo et al., 2021). To our knowledge, *Streptococcus pneumoniae* (*S. pneumoniae*) is the principal causative pathogen of bacterial pneumonia, otitis media, meningitis and septicemia (Engholm et al., 2017). However, currently developed vaccines could only protect against certain serotypes excluding the other concurrent invasive pathogens (Gonzalez-Miro et al., 2017). To tackle these challenges, González-Miro et al. fused a conserved antigen protein, PsaA, to PhaC, making the self-assembled PHB beads as an antigen presenting carrier, which could induce a significant and specific Th-2-type immune response (Gonzalez-Miro et al., 2017). Another serotype-independent antigen pneumolysin (Ply) is also studied and coated on PHB beads using similar strategy. Balb/c mice immunized with Ply-PhaC-modified PHB beads show higher IgG levels than the respective soluble counterparts, and the induced IgG antibodies could recognize Ply in whole cell lysates of six different serotypes of *S. pneumoniae* (Gonzalez-Miro et al., 2018).

Tuberculosis (TB) is an infectious disease caused by *Mycobacterium tuberculosis*, leading to approximately 2 million deaths and 10 million new cases each year globally (Kaufmann, 2010; Organization, 2015). PHB beads were engineered to display mycobacterial antigens, functioning as a vaccine against TB and immunodiagnostic reagents for the skin test of bovine TB diagnosis (Figure 4D) (Parlane et al., 2012; Chen et al., 2014b) (Parlane et al., 2016; Chen et al., 2019a). By fusing different target enzymes to PAPs, other PHA beads-based vaccines were developed to target at *Neisseria meningitidis* and Hepatitis C Virus (HCV) (Parlane et al., 2011; Lee et al., 2017). More interestingly, the natural MCL-PHA producer, *Pseudomonas aeruginosa*, was engineered to accumulate MCL-PHA beads coated with its own antigens (Gonzalez-Miro et al*.*, 2019). This ingenious idea implies that PHA beads with associated host cell proteins (HCPs) could be interpreted as a large antigenic repertoire in the future research (Lee et al., 2017). Except using as vaccines, recent efforts give evidence for the adjuvant potential of PHA bead by enhancing the immune response in the whole-killed *Pasteurella multocida* B:2 (PMB2) vaccinated rats (Mohamed et al., 2021).

It has been a long history since PHA nanoparticles were developed as the base of biosensors (Moradali and Rehm, 2020). For example, a sensitive and substrate-specific PHA/gold nanoparticles coated with peroxidase enzyme was developed to be an electrochemical biosensor for the determination of artemisinin level in human serum (Phukon et al., 2014); a functionalized PHA beads was developed by Kelwick et al. for protease detection (Figure 4E) (Kelwick et al., 2021), such as determining the proteolytic activity of AcTEV, cercarial elastase from the *Schistosoma mansoni*-derived cercarial transformation fluid samples, as well as cancer-associated metalloproteinases in extracellular vesicle and cell-conditioned media samples. These attempts and new findings demonstrate that PHA beads could be a promising platform of healthcare biosensors for the quantitative determination of varied proteases (Kelwick et al., 2021).

## Conclusion and Perspectives

PHA-based micro- and/or nano-carriers used as drug delivery systems have exhibited versatile therapeutic applications in BAS delivery, especially in providing innovative drug delivery strategies, based on micro-scale *in vitro* fabricated and nano-scale *in vivo* self-assembled PHA granules. This study summarizes advanced progresses of the biosynthesis, properties and fabrications and highlights therapeutic applications of PHA-based BAS delivery systems. Notably, compared with the PLA/PLGA particles, the self-assembled PHA beads have advantages of keeping the integrity of antigens and releasing beneficial degradation compound (3HB), making PHA particles biocompatible and favorable for clinical uses.

Many studies have reported that novel-type PHA containing units of non-natural structures, such as vinyl and azido PHA, have been synthesized using metabolically engineered microbes powered by system and synthetic biology, expanding the possibility of diverse chemical modification and providing new approaches for functional BAS delivery particle fabrication for customized and precise medical treatments in the future (Pinto et al., 2016; Yu et al., 2020a; Li et al., 2021b). Besides, the rapid developments of biotechnology have advanced the innovations of designs and fabrications of self-assembled PHA-based BAS carriers for therapeutic applications. For example, the deletion of *phaP* gene that encodes phasin (PhaP) protein involved in the regulation of PHA granule size, single granule PHA of larger and equal size can be obtained (Shen et al., 2019), which showed better stability and uniformity in medical uses compared to the naturally synthesized ones ranged from nano-to micro-scale.

Recently, some probiotics, such as *Bacillus subtilis* and *lactobacillus*, have been engineered to produce PHA of high production yield (Singh et al., 2009; Monilola and Makinde, 2020). Interestingly, Yan et al. showed proven success in colitis treatment by engineering the synthesis pathway of 3HB, the natural unit of PHB, in probiotic *Escherichia coli* Nissle 1917 (Yan et al., 2021). It is thus possible to construct different PHA synthetic pathways in other bacterial hosts of interest, especially in probiotics used for *in vivo* therapeutic, making PHA granules as versatile building blocks for various therapeutic applications in the coming future.

Moreover, since tumor-targeted bacteria have been utilized for oncotherapy by regulating the tumor microenvironment due to its proven effectiveness of antitumor and adaptive antitumor response (Zhou et al., 2018; Cao and Liu, 2020; Rong et al., 2020). Similarly, the genetically reprogramed bacteria able to target tumor cells and control the release of intracellular BAS packaged carriers, which are pre-accumulated PHA granules coated with *in vivo* biosynthesized antitumor agents, can significantly increase the drug response time for oncotherapy. Therefore, with the development of rational microbial engineering and profound studies of tailor-made PHA synthesis, probiotics pre-loaded with *in vivo* assembled PHA-based BAS carriers are becoming an alternative and sufficient solution in many therapeutic applications, including traditional drug delivery and synthetic live therapy. However, the safety of live bacteria therapy still remains a concern to be addressed.
